# Job demands and job control and their associations with disability pension—a register-based cohort study of middle-aged and older Swedish workers

**DOI:** 10.1007/s00420-023-01995-4

**Published:** 2023-07-14

**Authors:** Daniel Falkstedt, Melody Almroth, Tomas Hemmingsson, Angelo d’Errico, Maria Albin, Theo Bodin, Jenny Selander, Per Gustavsson, Katarina Kjellberg

**Affiliations:** 1 Institute of Environmental Medicine, Karolinska Institutet, Solnavägen 4, 10th Floor, 113 65 Stockholm, Sweden; 2 Centre for Occupational and Environmental Medicine, Region Stockholm, Stockholm, Sweden; 3 Department of Public Health Sciences, Stockholm University, Stockholm, Sweden; 4 Department of Epidemiology, Local Health Unit ASL TO 3, Grugliasco, Turin, Italy

**Keywords:** Occupational exposure, Psychosocial factors, Retirement benefits, Mental disorders, Musculoskeletal diseases, Cardiovascular diseases

## Abstract

**Objectives:**

Job demands and control at work and their combination, job strain, have been studied in relation to risk of disability pension (DP) previously. In the present study, based on registry data, we aimed to deepen the knowledge by analyzing major disease groups among the DPs, dose–response shape of the associations, and potential confounding effects of physical workload.

**Methods:**

Approximately 1.8 million workers aged 44 or older and living in Sweden in 2005 were followed up for 16 years, up to a maximum of 65 years of age. We linked mean values of job demands and job control, estimated in a job-exposure matrice (JEM) by gender, to individuals through their occupational titles in 2005. These values were categorized by rank order, and, for the construction of job-strain quadrants, we used a median cut-off. Associations with DP were estimated in Cox proportional-hazards models.

**Results:**

In models accounting for covariates including physical workload, low levels of job control were associated with higher risk of DP among both men and women. This association was most clear for DP with a psychiatric diagnosis, although a dose–response shape was found only among the men. High levels of job demands were associated with *decreased* risk of DP across diagnoses among men, but the same association varied from weak to non-existing among women. The high- and passive job-strain quadrants both showed increased risk of DP with a psychiatric diagnosis.

**Conclusion:**

The results suggest that, at the occupational level, low job control, but not high job demands, contributes to an increased incidence of DP, particularly regarding DP with a psychiatric diagnosis.

## Background

Poor work environments reduce workers’ opportunities for remaining in the workforce, both by causing ill health and by being an obstacle for those with existing health problems. Accordingly, there is evidence of associations between work-environmental factors and disability pension (Falkstedt et al. 2021; Knardahl et al. 2017; Pedersen et al. 2020), which is granted to workers with permanently reduced work ability. Disability pensions, however, are increasingly seen as problematic, because of aging populations, welfare systems under pressure and, thus, a perceived need for extended working lives (Hinrichs 2021).

Psychosocial factors at work could hypothetically contribute to the risk of disability pension. An influential construct for the study of psychosocial health effects at work is ‘job strain’, where ‘high strain’ is defined as the combination of low control and high demands in a job (Karasek and Theorell 1990). There is empirical support for its hypothesized effect on disability pensions, although there are inconsistencies. According to the most recently published review, (Knardahl et al. 2017) the combination of high demands and low job control seems to be a risk factor, but so does low control on its own. The latter was confirmed in two fairly recent Danish studies, based on self-reported data on work characteristics and a job-exposure matrix (JEM), respectively (Framke et al. 2020; Sundstrup et al. 2018). Mechanistically, the job-strain model is considered to measure mental strain with negative effects on both psychological and somatic outcomes. However, in line with the recent literature (Knardahl et al. 2017), we expect a higher complexity in relation to disability pension.

Associations between demands/control at work and major diagnoses behind disability pensions could provide clues to the impact of psychosocial working conditions on older workers’ ability to remain in work. However, these associations have been estimated in a limited number of studies, and often based on information about psychosocial factors at work reported by the study participants themselves. High strain has been shown to be associated with increased risk of disability pension, both with a depression diagnosis and a musculoskeletal diagnosis (Juvani et al. 2018), and in one study also with disability pension due to coronary heart disease (Mantyniemi et al. 2012). Low control at work has, in several studies (Lahelma et al. 2012; Leineweber et al. 2019; Samuelsson et al. 2013), been shown to be associated with disability pension with a psychiatric diagnosis. Additionally, both association (Lahelma et al. 2012) and absence of association (Leineweber et al. 2019) between low job control and disability pension with a musculoskeletal diagnosis have been shown. Lastly, high demands have been shown to be associated both with higher (Lahelma et al. 2012; Leineweber et al. 2019) and with lower (Samuelsson et al. 2013) relative risk of disability pension with a psychiatric diagnosis, and with lower (Ropponen et al. 2013) or uncertain (Leineweber et al. 2019) relative risk of disability pension with a musculoskeletal diagnosis.

Evidence of dose–response relationships strengthens inference, but only a few previous studies examined dose–response (Emberland et al. 2017; Ropponen et al. 2013; Sundstrup et al. 2018), and this was not done in women and men separately or in relation to disability pensions divided into diagnostic categories other than musculoskeletal disorders. Moreover, study populations in previous studies usually represented a limited part of the labor market and, thus, the entire occupational exposure range was not captured in the studies. A gradually increased risk of disability pension with a musculoskeletal diagnosis has been shown in relation to low control at work (Ropponen et al. 2013).

Associations between job-strain components and diagnosis-specific disability pension may be confounded by differences in physical workload, as a higher physical workload can be expected in occupations characterized by lower control at work and/or lower job demands. A limited number of previous studies included physical workload as a covariate (Emberland et al. 2017; Lahelma et al. 2012; Sundstrup et al. 2018). However, the significance of this potential confounding is unknown for associations with specific diagnoses behind disability pension.

The aim of this study was to gain a more detailed understanding of job demands and control at work in relation to the prospective risk of disability pension. We examined hypothetical dose–response relationships with all-cause and cause-specific disability pension using job-exposure matrices for classification of job demands, job control, job strain, and (as a potential confounding factor) physical workload. All middle-aged and older men and women with a registered occupation in Sweden were included in the study and, thus, the full range of occupations was covered.

## Methods

### Study population, setting, and design

The study was based on the recently established Swedish Work, Illness, and Labor-Market Participation (SWIP) Cohort, which includes all individuals 16–64 years of age and registered as living in Sweden in 2005 (Almroth et al. 2021; d'Errico et al. 2022; Falkstedt et al. 2021). In the study, we focused on the population of 1,838,365 women and men born during the years 1942–1961 and having an occupational code in the year 2005. This year was the baseline year of the study, and follow-up regarding date and diagnosis of disability pension was made prospectively for the years 2006 to mid-2020. At baseline, the individuals in the study were aged 44–63 years.

The Regional Ethics Review Board in Stockholm, Sweden, approved the research (protocol no. 2017/1224-31 and no. 2018/1675-32).

### Data collection and variables

Data on occupations, classified on a four-digit level according to SSYK96 (Sweden’s classification of occupations based on ISCO-88), were obtained from Statistics Sweden’s ‘LISA’ register (‘Longitudinal integrated database for health insurance and labor market studies’) (Ludvigsson et al. 2019) The register starts in the year 1990 and contains information on every individual in Sweden aged 16–64, but complete occupational data are available only from 2005. The occupational data were linked to the cohort and used for classification of work-environmental exposures, separately for men and women. Further data, used for the analyses, were obtained from the LISA register, the Total Population Register at Statistics Sweden (Ludvigsson et al. 2016), the Population and Housing Censuses of 1960 and 1970, and the Swedish Social Insurance Agency’s MIDAS register (‘Micro Data for Analysis of the Social Insurance System’), (forsakringskassan.se [Internet],^ 2023^a).

#### Job demands, job control, and job strain (exposures)

Levels of job demands and control at work were assigned to individuals using a Swedish JEM. In this JEM, mean scores have been calculated for each occupation based on item scores from around 90,000 participants in the Swedish Work Environment Surveys during the years 1997–2013. Job demands are measured using three questions focused on stress, time pressure, and requirements for attention and concentration in the work. Job control is measured based on four questions measuring decision authority and, more specifically, the possibilities to influence which tasks to do, the pace of work, when to take breaks, and the structure of the work. Details of the construction and its validity are described in a previous publication (Fredlund et al. 2000), but external validation has not been possible. However, results in several recent studies using this JEM (Almroth et al. 2021; Almroth et al. 2022a, b) were consistent with similar studies from other countries.

Assignment of mean scores for job demands and job control to individuals were made by using the individuals’ occupational codes (SSYK96) from the year 2005. Subsequently, these values were categorized according to their quintile distribution (separately for men and women), resulting in five categories ranging from low to high (‘low’, ‘medium low’, ‘medium’, ‘medium high’, and ‘high’). Job strain was constructed as the combination of decision authority and job demands split at their respective median and was categorized into four mutually exclusive categories: low strain jobs (low demands and high decision authority); passive jobs (low demands and low decision authority); active jobs (high demands and high decision authority); and high strain jobs (high demands and low decision authority).

#### Disability pension (outcome)

In Sweden, disability pension is a compensation for those between ages 30 and 64 who due to illness, injury or disability will probably never be able to work full time (forsakringskassan.se [Internet], 2023b). There must be a proven reduction in work ability by at least one fourth in relation to jobs available on the labor market, including jobs that are arranged for persons with disabilities, such as employment with salary grants. Depending on how much the work ability is reduced, 100%, 75%, 50%, or 25% disability pension can be paid. The Social Insurance Agency may replace a sickness absence benefit with disability pension on its own initiative, but it is otherwise applied for. If a disability pension is granted, it can cover up to about 65% of lost income in those with previous employment.

Data on disability pension (e.g., granting date, grade, and ICD diagnosis) were obtained from the above-mentioned database MiDAS. Virtually all reimbursements related to disability pension are registered in this database. In the present study, disability pension diagnoses (according to ICD, 10th version) were as follows: musculoskeletal diagnoses, codes M00-M99; psychiatric diagnoses, codes F00-F99; and circulatory system diagnoses, codes I00-I99.

All first-time disability pensions (both full and partial) post baseline were included in the study, based on an assumption that they all capture reduced ability to work fully.

#### Covariates

Level of education, civil status, being born abroad, and history of unemployment were variables based on data obtained from the above-mentioned administrative registers (Ludvigsson et al. 2019). These variables may covary with demands/control and are also assumed to jointly capture differences in risk of disability pension during the follow-up. *Level of education* consisted of four groups based on number of years of education: ≤ 9, 10–11, 12, and ≥ 13 years, which corresponds to lower secondary or less, vocational higher secondary, academic higher secondary, and university-level studies. *Civil status* was coded as married (or registered partner), unmarried/single, divorced, widow(er). *Being born abroad* was based on country of birth and was dichotomized to indicate whether the person was born in Sweden or not. *History of unemployment* was calculated from the total number of days with benefits during five years before the baseline (2000–2004) and was divided into (1) no unemployment days, (2) 365 or fewer days of unemployment, and (3) more than 365 days of unemployment.

*Socioeconomic position during the index person’s childhood/adolescence* was obtained by linking the index person to their parents’ census information from 1960 or 1970. The parents’ socioeconomic position was classified as high-skilled non-manual employees at a higher level, intermediate non-manual employees, assistant non-manual employees, skilled manual workers, non-skilled manual workers, farmers, or those with no occupation reported.

The study participants’ degree of overall *physical workload* was classified using a JEM also based on data from the Swedish Work Environment Surveys 1997–2013 (Badarin et al. 2021). A gender-specific mean score for overall physical exposure, based on eight questions regarding physical loads that involve heavy lifting, uncomfortable working postures, repetitive work, and physical demanding work, was obtained from the matrix, and linked to individuals through data on their occupations in 2005.

### Statistical analysis

Individuals who already had a disability pension in 2005 were excluded (*N* = 155,425), as were those among the remaining individuals who lacked information on any of the control variables (*N* = 10,386); the reason for limited missingness is that all individuals with a registered occupation receive a value of job demands, job control, and physical workload. The number of men and women included in all analyzes and regression models was therefore 840,620 and 831,555, respectively.

To characterize the male and female cohort members with different degrees of job demands and job control at baseline, we calculated the distribution of the covariates across the exposure quintiles.

To examine whether and how high levels of job demands, low levels of job control, and their combination (high strain) may increase the risk of disability pension, we used Cox proportional-hazards regression, which estimates hazard ratios (HR). Person-time was calculated from January 1, 2006, up to the date of emigration, death, disability pension or the end of follow up on June 30, 2020. Associations with disability pensions due to any diagnosis or a psychiatric, cardiovascular, or a musculoskeletal diagnosis were analyzed in crude and covariate-adjusted regression models, and separately for women and men.

In most cases, a disability pension is preceded by long-term sickness absence, and due to this structural relation, sickness absence before baseline was not included as a covariate. However, as a sensitivity analysis, we estimated the associations on a population excluding individuals with sickness absence registered at the Swedish Social Insurance Agency between 2001 and 2005.

## Results

Men with lower job demands and lower job control were on average younger, more likely to have parents in a manual socioeconomic position in childhood, more likely to be foreign born, much more likely to have compulsory education only, more likely not to be married (i.e. unmarried, divorced or widowed), more likely to have days in unemployment before baseline, and also much more likely to be exposed to heavy physical workload, as compared with men with higher job demands and higher job control, respectively (Table 1, upper section). Women showed a similar pattern (Table 1, lower section), although age and, also, the likelihood of not being married deviated from this.

**Table 1:** Covariate distributions across degrees of job demands and job control, measured by job-exposure matrices

	Job demands					Job control				
	Low	Mid/low	Mid	High/mid	High	High	High/mid	Mid	Mid/low	Low
Men										
*N*	164,725	164,034	172,737	168,892	170,232	171,530	175,029	160,848	176,143	157,070
Age > 52 years (> median)^a^, %	45.48	46.11	47.09	50.29	50.76	50.36	50.67	47.15	46.56	44.75
Manual SEP origin^b^, %	69.27	64.40	51.73	53.27	42.35	40.48	52.99	63.57	63.60	60.34
Foreign born, %	16.51	10.32	8.17	8.16	9.38	6.28	6.81	11.01	11.32	17.61
Compulsory education only^c^, %	35.88	29.39	15.56	21.40	10.53	8.46	20.05	25.40	31.59	26.83
Not married^d^, %	48.09	42.90	37.63	37.90	29.20	29.26	35.61	42.15	44.61	44.19
Pre-baseline unemployment^e^, %	22.09	18.39	11.48	16.12	9.67	8.07	13.44	18.42	19.63	18.14
Heavy physical workload^f^, %	31.18	44.34	19.46	0.78	2.82	1.85	12.49	38.67	15.86	30.98
Women										
*N*	158,009	163,947	160,856	176,132	172,611	177,066	163,349	156,112	198,382	136,646
Age > 52 (median)^a^, %	48.61	50.97	43.39	44.46	47.86	48.05	49.93	45.92	43.54	48.60
Manual SEP origin^b^, %	64.94	62.58	61.34	50.09	42.44	47.61	56.25	55.71	63.88	54.45
Foreign born, %	15.51	12.52	11.23	8.74	9.14	8.57	11.72	10.55	13.21	12.66
Compulsory education only^c^, %	31.57	24.63	8.40	8.56	2.31	10.23	18.81	15.14	11.86	19.63
Not married^d^, %	42.51	40.86	39.04	39.37	35.27	38.74	40.31	39.58	40.19	37.48
Pre-baseline unemployment^e^, %	19.96	20.71	14.53	11.54	8.40	11.80	16.07	14.19	16.87	15.31
Heavy physical workload^f^, %	34.41	11.19	44.70	7.81	1.89	1.76	18.75	12.02	37.76	25.05

^a^At baseline

^b^Head of household in skilled or unskilled work during index person’s childhood

^c^Nine years or less

^d^Not married (incl. legal partner)

^e^Any unemployment during 2000–2004

^f^In quintile with highest exposure scores

During the follow-up period from 2006 to mid-2020, 44,255 men (5.3%) and 71,140 women (8.6%) were granted a disability pension. Of these, 10,199 men and 21,116 women had a psychiatric diagnosis as the main diagnosis, 7029 men and 4538 women had a CVD diagnosis as the main diagnosis, and 11,452 men and 24,612 women had a musculoskeletal diagnosis as the main diagnosis.

High levels of job demands were associated with a clearly *decreased* risk of receiving disability pension in the crude (age-adjusted only) model (Table 2). For men, this pattern was consistent across all types of disability pension (highest level of job demands and any diagnosis, HR = 0.41; psychiatric diagnosis, HR = 0.54; CVD diagnosis, HR = 0.46; musculoskeletal diagnosis, HR = 0.23), while for women disability pensions with a psychiatric diagnosis seemed to deviate slightly from it (any diagnosis, HR = 0.57; psychiatric diagnosis, HR = 0.87; CVD diagnosis, HR = 0.56; musculoskeletal diagnosis, HR = 0.32); for both men and women, the inverse association between job demands and disability pension was particularly strong regarding disability pensions with a musculoskeletal diagnosis. In models adjusting for pre-baseline covariates (model II) and, in addition to that, physical workload (model III), the associations were gradually attenuated, and none showed a remaining dose–response shape. For the women, the associations were almost or completely eliminated after the adjustments.

**Table 2:** Hazard ratios and 95% confidence intervals for job demands and disability pension diagnosis (any, psychiatric, cardiovascular, or musculoskeletal)

	Men										Women									
DP diagnosis		Model I			Model II			Model III				Model I			Model II			Model III		
Job demands	Cases	HR	95%CI		HR	95%CI		HR	95%CI		Cases	HR	95%CI		HR	95%CI		HR	95%CI	
Any																				
Low	13,633	1.00			1.00			1.00			16,880	1.00			1.00			1.00		
Mid/low	9598	0.69	0.68	0.71	0.76	0.74	0.78	0.76	0.74	0.78	16,109	0.91	0.89	0.93	0.95	0.93	0.97	0.99	0.97	1.01
Mid	7284	0.50	0.48	0.51	0.63	0.61	0.65	0.75	0.73	0.78	14,812	0.86	0.84	0.88	0.95	0.93	0.98	0.91	0.89	0.94
High/mid	7725	0.54	0.52	0.55	0.65	0.63	0.67	0.81	0.78	0.83	12,541	0.65	0.64	0.67	0.83	0.81	0.85	0.95	0.93	0.98
High	6015	0.41	0.40	0.43	0.57	0.55	0.59	0.75	0.72	0.77	10,798	0.57	0.55	0.58	0.81	0.79	0.83	0.93	0.91	0.96
Psychiatric																				
Low	3178	1.00			1.00			1.00			4315	1.00			1.00			1.00		
Mid/low	1775	0.56	0.53	0.59	0.60	0.57	0.64	0.60	0.57	0.64	4219	0.95	0.91	0.99	0.96	0.92	1.00	0.99	0.95	1.04
Mid	1686	0.50	0.47	0.53	0.58	0.54	0.61	0.62	0.59	0.66	4063	0.91	0.87	0.95	0.97	0.93	1.01	0.99	0.94	1.03
High/mid	1817	0.56	0.53	0.60	0.61	0.58	0.65	0.67	0.63	0.71	4356	0.89	0.85	0.93	0.95	0.91	1.00	1.02	0.97	1.07
High	1743	0.54	0.51	0.57	0.61	0.58	0.65	0.69	0.65	0.73	4163	0.87	0.84	0.91	0.94	0.90	0.99	1.01	0.96	1.06
CVD																				
Low	2010	1.00			1.00			1.00			1108	1.00			1.00			1.00		
Mid/low	1464	0.72	0.68	0.77	0.79	0.74	0.84	0.80	0.74	0.85	1084	0.93	0.86	1.01	0.96	0.89	1.05	1.00	0.91	1.09
Mid	1192	0.56	0.52	0.60	0.72	0.67	0.77	0.82	0.76	0.88	892	0.81	0.74	0.88	0.89	0.82	0.97	0.87	0.80	0.95
High/mid	1381	0.65	0.61	0.70	0.81	0.75	0.87	0.94	0.88	1.01	767	0.63	0.58	0.69	0.83	0.76	0.91	0.92	0.83	1.01
High	982	0.46	0.42	0.49	0.67	0.61	0.72	0.81	0.74	0.88	687	0.56	0.51	0.62	0.88	0.80	0.98	0.97	0.87	1.07
MSD																				
Low	3953	1.00			1.00			1.00			6808	1.00			1.00			1.00		
Mid/low	3048	0.77	0.73	0.80	0.86	0.82	0.90	0.86	0.82	0.90	6264	0.88	0.85	0.91	0.92	0.89	0.95	0.98	0.95	1.02
Mid	1734	0.41	0.39	0.43	0.58	0.55	0.61	0.78	0.74	0.83	5637	0.83	0.80	0.86	0.95	0.92	0.99	0.86	0.83	0.89
High/mid	1730	0.41	0.39	0.44	0.55	0.52	0.59	0.80	0.76	0.85	3487	0.46	0.44	0.48	0.70	0.67	0.73	0.91	0.87	0.95
High	987	0.23	0.22	0.25	0.39	0.36	0.42	0.64	0.59	0.68	2416	0.32	0.31	0.34	0.64	0.61	0.67	0.85	0.80	0.89

*DP* disability pension, *CVD* cardiovascular disease, *MSD* musculoskeletal disorder, *HR* hazard ratio, *95%CI* 95% confidence interval

Model I) adjusted for age; II) also adjusted for childhood SEP, being foreign born, level of education, civil status, and days in unemployment the years before baseline; III) also adjusted for occupational physical workload

Low levels of job control (Table 3, crude model) were associated with an increased risk of disability pension overall during the follow-up period (lowest level of job control: men, HR = 2.64; women, HR = 1.83), as well as disability pensions with a psychiatric (men, HR = 2.29; women, HR = 1.54), cardiovascular (men, HR = 2.33; women, HR = 1.46), or musculoskeletal diagnosis (men, HR = 4.60; women, HR = 2.66). This association showed a dose–response pattern for men, except for disability pension with a musculoskeletal diagnosis. For women, there was not a dose–response pattern, as the medium low job control category showed the highest hazard ratios for all outcomes. All crude estimates were substantially attenuated after adjustment for covariates in model II; the attenuating effect was particularly strong for the men. The estimates were further attenuated after adjustment for differences in physical workload (model III), except the estimates for disability pension with a psychiatric diagnosis. In this case, an opposite effect from adjustment was seen.

**Table 3:** Hazard ratios and 95% confidence intervals for job control and disability pension diagnosis (any, psychiatric, cardiovascular, or musculoskeletal)

	Men										Women									
DP diagnosis		Model I			Model II			Model III				Model I			Model II			Model III		
Any	Cases	HR	95%CI		HR	95%CI		HR	95%CI		Cases	HR	95%CI		HR	95%CI		HR	95%CI	
Job control																				
Low	10,638	2.64	2.55	2.74	2.00	1.93	2.07	1.66	1.60	1.73	12,281	1.83	1.78	1.88	1.70	1.65	1.75	1.36	1.32	1.40
Mid/low	11,498	2.53	2.45	2.62	1.89	1.83	1.96	1.58	1.52	1.64	21,752	2.27	2.22	2.33	1.97	1.92	2.02	1.48	1.44	1.53
Mid	10,011	2.40	2.32	2.49	1.83	1.77	1.90	1.53	1.47	1.59	14,517	1.89	1.85	1.95	1.79	1.75	1.84	1.50	1.45	1.54
High/mid	7580	1.65	1.59	1.71	1.41	1.36	1.46	1.33	1.28	1.38	13,670	1.69	1.65	1.74	1.52	1.48	1.56	1.38	1.34	1.42
High	4528	1.00			1.00			1.00			8920	1.00			1.00			1.00		
Psychiatric																				
Low	2597	2.29	2.12	2.47	1.99	1.86	2.14	2.11	1.81	2.47	3471	1.54	1.46	1.63	1.48	1.40	1.55	1.53	1.34	1.74
Mid/low	2530	1.90	1.76	2.06	1.81	1.69	1.94	1.90	1.62	2.22	5916	1.70	1.62	1.78	1.73	1.65	1.81	1.77	1.55	2.01
Mid	2158	1.84	1.71	1.99	1.71	1.59	1.84	1.99	1.70	2.32	4484	1.74	1.65	1.83	1.68	1.60	1.76	1.75	1.54	1.98
High/mid	1701	1.33	1.22	1.44	1.35	1.25	1.45	1.55	1.33	1.82	4279	1.57	1.49	1.65	1.53	1.46	1.60	1.69	1.51	1.91
High	1213	1.00			1.00			1.00			2966	1.00			1.00			1.00		
CVD																				
Low	1603	2.33	2.13	2.55	1.72	1.58	1.88	1.41	1.18	1.67	760	1.46	1.31	1.63	1.36	1.23	1.51	1.14	0.92	1.42
Mid/low	1836	2.31	2.12	2.53	1.69	1.55	1.84	1.35	1.15	1.60	1330	1.82	1.65	2.00	1.52	1.39	1.67	1.24	1.01	1.52
Mid	1488	2.07	1.89	2.27	1.51	1.38	1.65	1.17	0.98	1.39	862	1.49	1.34	1.65	1.38	1.25	1.53	1.29	1.05	1.59
High/mid	1318	1.64	1.49	1.80	1.36	1.24	1.48	1.22	1.04	1.44	914	1.44	1.29	1.60	1.30	1.18	1.44	1.28	1.05	1.56
High	784	1.00			1.00			1.00			672	1.00			1.00			1.00		
MSD																				
Low	2838	4.60	4.20	5.03	2.91	2.68	3.16	1.79	1.46	2.20	4538	2.66	2.52	2.81	2.29	2.18	2.41	1.16	1.00	1.34
Mid/low	3137	4.37	4.00	4.77	2.74	2.52	2.97	1.77	1.45	2.17	8512	3.47	3.30	3.65	2.61	2.49	2.74	1.36	1.18	1.57
Mid	2919	4.56	4.17	4.99	2.83	2.60	3.07	1.76	1.44	2.16	4764	2.50	2.37	2.64	2.19	2.08	2.30	1.43	1.23	1.65
High/mid	1856	2.57	2.34	2.82	1.90	1.74	2.07	1.60	1.30	1.97	4523	2.11	2.00	2.23	1.78	1.69	1.87	1.34	1.17	1.54
High	702	1.00			1.00			1.00			2275	1.00			1.00			1.00		

*DP* disability pension, *CVD* cardiovascular disease, *MSD* musculoskeletal disorder, *HR* hazard ratio, *95%CI* 95% confidence interval

Model I) adjusted for age; II) also adjusted for childhood SEP, being foreign born, level of education, civil status, and days in unemployment the years before baseline; III) also adjusted for occupational physical workload

For men, both high-strain (HR = 1.19) and passive jobs (HR = 1.45) showed increased risk of disability pension overall and due to a psychiatric (high-strain, HR = 1.55; passive, HR = 1.69) or a cardiovascular diagnosis (high-strain, HR = 1.22; passive, HR = 1.41), compared to low strain jobs (Table 4, crude model). For women, passive jobs were associated with an increased risk of disability pension across diagnoses (any diagnosis, HR = 1.27; psychiatric diagnosis, HR = 1.09; CVD diagnosis, HR = 1.19; musculoskeletal diagnosis, HR = 1.50) whereas high-strain jobs were associated with an increased risk due to psychiatric diagnosis only (HR = 1.10). Active jobs were associated with a lower risk of disability pension in both men and women, particularly disability pensions with a musculoskeletal diagnosis (men, HR = 0.34; women, HR = 0.44). After adjustments (models II and III), high-strain jobs and passive jobs remained associated with disability pensions with a psychiatric diagnosis in both men and women and with disability pensions due to CVD in men. Also, active jobs remained associated with a lower risk of disability pension with a musculoskeletal disorder in both men and women, and with a lower risk of disability pension with a psychiatric disorder in women.

**Table 4:** Hazard ratios and 95% confidence intervals for job strain (quadrants) and disability pension diagnosis (any, psychiatric, cardiovascular, or musculoskeletal)

	Men										Women									
DP diagnosis		Model I			Model II			Model III				Model I			Model II			Model III		
Job strain	Cases	HR	95%CI		HR	95%CI		HR	95%CI		Cases	HR	95%CI		HR	95%CI		HR	95%CI	
Any																				
Low strain	7605	1.00			1.00			1.00			17,397	1.00			1.00			1.00		
Active	8602	0.61	0.59	0.63	0.71	0.69	0.74	0.89	0.86	0.92	13,416	0.62	0.61	0.63	0.75	0.74	0.77	0.86	0.83	0.88
Passive	19,193	1.45	1.41	1.49	1.30	1.26	1.33	1.22	1.19	1.26	27,344	1.27	1.25	1.30	1.22	1.20	1.24	1.01	0.98	1.03
High strain	8855	1.19	1.15	1.23	1.17	1.13	1.20	1.18	1.14	1.22	12,983	0.86	0.84	0.88	1.13	1.10	1.15	1.05	1.02	1.08
Psychiatric																				
Low strain	1440	1.00			1.00			1.00			4846	1.00			1.00			1.00		
Active	2259	0.87	0.81	0.93	0.92	0.87	0.99	0.97	0.90	1.04	4816	0.80	0.77	0.83	0.84	0.81	0.88	0.88	0.85	0.92
Passive	4323	1.69	1.59	1.80	1.59	1.50	1.69	1.55	1.46	1.65	6748	1.09	1.05	1.13	1.09	1.05	1.13	1.08	1.03	1.12
High strain	2177	1.55	1.45	1.65	1.42	1.33	1.52	1.40	1.31	1.50	4706	1.10	1.06	1.15	1.15	1.10	1.20	1.12	1.07	1.17
CVD																				
Low strain	1186	1.00			1.00			1.00			1162	1.00			1.00			1.00		
Active	1524	0.69	0.64	0.75	0.83	0.77	0.90	0.98	0.90	1.07	932	0.67	0.61	0.73	0.85	0.78	0.92	0.93	0.85	1.02
Passive	2889	1.41	1.32	1.51	1.25	1.17	1.34	1.18	1.10	1.27	1694	1.19	1.10	1.28	1.12	1.04	1.21	0.97	0.90	1.06
High strain	1430	1.22	1.13	1.32	1.24	1.15	1.34	1.23	1.14	1.33	750	0.76	0.69	0.83	1.08	0.98	1.19	1.00	0.90	1.10
MSD																				
Low strain	2354	1.00			1.00			1.00			6294	1.00			1.00			1.00		
Active	1482	0.34	0.32	0.36	0.43	0.41	0.46	0.72	0.68	0.78	3346	0.44	0.42	0.46	0.63	0.60	0.66	0.84	0.80	0.88
Passive	5502	1.28	1.41	1.14	1.08	1.19	1.05	1.00	1.11	1.28	11,562	1.50	1.46	1.55	1.38	1.34	1.42	0.95	0.91	0.98
High strain	2114	0.91	0.86	0.96	0.93	0.87	0.98	0.98	0.92	1.04	3410	0.63	0.61	0.66	1.11	1.06	1.16	0.97	0.93	1.02

*DP* disability pension, *CVD* cardiovascular disease, *MSD* musculoskeletal disorder, *HR* hazard ratio, *95%CI* 95% confidence interval

Low strain: low demands & high control; Active: high demands & high control; Passive: low demands & low control; High strain: high demands & low control

Model I) adjusted for age; II) also adjusted for childhood SEP, being foreign born, level of education, civil status, and days in unemployment the years before baseline; III) also adjusted for occupational physical workload

Excluding individuals having any registered sickness absence in the years up to the baseline year did not attenuate the associations (results not shown). Sickness absence was excluded as a covariate in the main analyses because separation of disability pension and a preceding period of sickness absence may be questionable.

## Discussion

In summary, the covariate-adjusted analyses showed that low levels of job control were associated with higher risk of disability pension in a dose–response manner among men but not among women. High levels of job demands were associated with *lower* risk of disability pension across diagnosis categories among men but, among women, this lower risk only remained for disability pensions with a musculoskeletal diagnosis in fully adjusted models. Additionally, the high-strain and passive job quadrants—both including low job control—showed increased risk of disability pension with a psychiatric diagnosis, especially among the men.

### Comparison with previous studies

The association between job demands and disability pension, which differed between the men and the women, confirm a contradictory picture from previous studies (Juvani et al. 2018; Lahelma et al. 2012; Leineweber et al. 2019; Mantyniemi et al. 2012; Ropponen et al. 2013; Samuelsson et al. 2013; Sundstrup et al. 2018). For instance, high demands have been linked with both higher (Lahelma et al. 2012; Leineweber et al. 2019) and lower (Samuelsson et al. 2013) risk of disability pension with a psychiatric diagnosis, and with lower (Ropponen et al. 2013) or uncertain (Leineweber et al. 2019) risk of disability pension with a musculoskeletal diagnosis. The association between low job control and increased risk for disability pension, on the other hand, is in agreement with a more uniform picture (Knardahl et al. 2017). This increased risk has been seen for disability pension both with a psychiatric diagnosis and with a musculoskeletal diagnosis (Lahelma et al. 2012; Leineweber et al. 2019; Ropponen et al. 2013; Samuelsson et al. 2013), quite similar to the present study. In line with this, our results also confirm a picture from previous studies that job-strain quadrants that include low job control (high strain and passive jobs) tend to be associated with increased disability pension risks (Knardahl et al. 2017; Leineweber et al. 2019; Sundstrup et al. 2018).

The association between low job control and disability pension overall and, also, for disability pension with a psychiatric diagnosis showed dose–response patterns among men, but not among women. Such dose–response has previously been shown for low job control and disability pension with a musculoskeletal diagnosis (Ropponen et al. 2013), but this was not confirmed in the present study. However, our use of more exposure categories can make the studies difficult to compare directly. Further, no dose–response patterns were observed for the associations between lower job demands and higher risks of disability pension in our fully adjusted models. Similar results were shown in the few studies that examined this previously (Emberland et al. 2017; Ropponen et al. 2013; Sundstrup et al. 2018).

Control for potential confounders had a major attenuating effect on the associations, both for job demands and job control. This was seen in many previous studies as well (Juvani et al. 2018; Lahelma et al. 2012; Laine et al. 2009; Mantyniemi et al. 2012; Norberg et al. 2020; Ropponen et al. 2013), though not all (Leineweber et al. 2019). In our study, low job demands showed a modest (among men) or even eliminated (among women) association after adjustments, especially regarding disability pensions with a cardiovascular diagnosis. For disability pension with a psychiatric diagnosis, however, control for physical workload attenuated the association to a limited extent. On the other hand, control for physical workload had a particularly strong attenuating effect on the associations with disability pension with a musculoskeletal diagnosis. An earlier Finnish study showed similar results (Lahelma et al. 2012).

The present study contributed beyond previous studies with several results. We showed that high job demands for both men and women lacked a dose–response association with disability pension regardless of diagnosis group; that low job control for men but not women showed dose–response associations with different causes of disability pension; and that disability pension with a psychiatric diagnosis presented the strongest association in models with comprehensive covariate adjustment. Additionally, we showed that passive jobs and high-strain jobs, which both include low job control as a component, showed associations with disability pension with a psychiatric diagnosis for men as well as women, and for men also an association with disability pension with a cardiovascular diagnosis.

### Interpretations of the results

A decision at the Swedish Social Insurance Agency to grant a disability pension is based on an assessment of full or partial long-term reduction of the individual’s work ability (forsakringskassan.se [Internet], 2023a). In turn, work ability is a function of both health and capacity of the individual and of the requirements and conditions in the individual’s work.

As we see it, there are two main explanatory models, which are not mutually exclusive, with relevance to the results as follows:

First, there is the hypothesis of high job demands and low job control as work-related stress mechanisms with negative effects on psychological and somatic wellbeing and health (Karasek and Theorell 1990); the risk of disability pension is affected to the extent that various forms of ill-health are affected by these conditions at work. Evidence for negative effects on psychological and physical health was recently summarized in a review of reviews by Niedhammer et al. (2021). This review pointed in the direction of effects on a number of different health outcomes, not least with regard to coronary heart disease, depression, and musculoskeletal disorders.

We recently published studies of job-strain components and depression, alcohol-related morbidity, and suicidal events in the total population of Swedish workers. The results of these studies were in support of an effect of low control at work on risks of mental health (Almroth et al. 2021; Almroth et al. 2022a, b). However, the results were also in support of increased risks of mental health in occupations with low job demands, rather than in occupations with high job demands.

A second hypothesis is that high job demands and low control at work also have negative effects on the opportunities for participation in work for older workers with various forms of health problems and reduced work capacity (Gragnano et al. 2018). This contributes to a higher complexity in the associations, where demands and control interact with both biological and psychological factors in individuals as well as social factors around them. Previous studies in the field have usually only reported an association with the risk of disability pension overall (Knardahl et al. 2017), which provides a limited basis for discussing different interpretations and explanations. The results of the present study indicate that it would be low control at work rather than high job demands that could have such an effect. An association across diagnoses, seen for low levels of control at work and disability pension, is in line with the hypothesis.

The inverse associations between job demands and risk of disability pension shown in the study may seem counterintuitive. However, one possibility is that, at the occupational level, these associations capture a preventive effect of greater variety in tasks, more room for creativity and greater commitment in jobs with high demands. Nevertheless, residual confounding from social class is also a possible explanation for the associations, as level of education had to be used as a proxy variable in the regression models.

Statistical control for confounding factors, measured from childhood and onwards, yielded results that support explanations through selection effects for some of the associations of job demands, job control, and job-strain components with disability pension. It is well known that workers with a low level of education and periods of previous unemployment are a group with a weaker position in the labor market and with greater difficulties in finding new employment. Also, it has been shown in European countries that the gap in work participation between workers with and without chronic diseases is largest among those with a short education (Schram et al. 2019).

Statistical control for differences in physical workload also had an attenuating effect on the associations in the present study, although increased risks of disability pensions with cardiovascular and musculoskeletal diagnoses remained for both high job demands and low job control, especially regarding the men. Admittedly, the adjustment across the disability pension diagnoses was more exploratory and pragmatic than theoretically driven. Nonetheless, we have shown previously that heavy physical workload is important in relation to disability pensions (Falkstedt et al. 2021) and, clearly, it should be taken into account in future studies of occupational psychosocial working conditions and disability pension.

Both crude and multivariable models showed stronger and more dose–response-like associations between job demands, job control, job strain, and disability pensions for the men than for the women. On the other hand, all the outcomes except disability pension with a cardiovascular diagnosis had a higher incidence among the women. It could be that, because of more disability pensions not related to work among the women, the association estimates for the women were not as ‘clean’ and therefore weaker. It could also be that the occupations of the men and the women in the study were overall too different to produce comparable estimates of the associations; there is a clear gender imbalance for major occupations in Sweden, for example in healthcare.

### Strengths and limitations

The size of the study population was larger than in all other previous studies that examined similar research questions. The basis for this was the use of register data, which also greatly reduces external and internal information loss. It follows that we could provide analyzes with unusual precision for the associations with disability pensions with a psychiatric, cardiovascular, or musculoskeletal diagnosis, stratified by gender. It also follows that we covered the entire range of occupational exposure to job demands and job control on the labor market and, thus, had an unusually good basis for the examination of dose–response shape of associations. A further strength was the use of JEMs to rank all male and female workers in Sweden in terms of these psychosocial occupational exposures and, also, physical workload. Potentially, the utilization of JEMs prevents differential misclassification and biased risk estimates that could have resulted if data on work-environment factors reported by the individuals themselves had been used. However, whether independent and ‘objective’ or ‘subjective’ measurement of the psychosocial work environment provides the best conditions for relevant and unbiased effect estimation constitutes a continuous discussion (Kompier 2005). An additional strength was the ability to account for covariation between job demands, job control, and physical workload at the occupational level. A limitation then was that job control had to be measured with decision authority items only, because ‘skill discretion’ in our demands/control JEM has items overlapping with physical workload, such as monotony. However, measuring job control in this way is not uncommon (Theorell 2020).

Using JEMs also has disadvantages. Through its design, a JEM eliminates individual differences in exposure within occupations, which can result in non-differential misclassification and downward-biased risk estimates. There are validation studies showing that this could affect the measurement of job demands more, compared to job control (Niedhammer et al. 2008; Solovieva et al. 2014). Self-reported job demands and job control would capture differences within occupations to a larger extent but could only be collected in a much smaller study population. On the positive side, both recent (Madsen et al. 2018) and older studies (Bosma et al. 1997; Theorell et al. 1998) on psychosocial and physical workload factors have been shown to give comparable results when using job-exposure matrices and self-reported exposures side by side. The restriction to baseline occupational data used for assignment of work-environment exposures may also have caused a certain degree of exposure misclassification during the follow up. However, we know from our own computations (not shown) that the proportion of middle-aged and older workers changing occupations tends to be small. Accordingly, we assume that we mostly capture outcomes of long-term exposure in the study.

We did not have access to data on differences in lifestyle-related risk factors, such as tobacco smoking, physical inactivity, and obesity and, thus, we cannot rule out unmeasured confounding. It is sometimes difficult to determine the effect of control for such factors in previous studies (Juvani et al. 2018; Sundstrup et al. 2018), but in a few of these (Canivet et al. 2013; Laine et al. 2009) the effect appeared to be limited. Also, we presume that the adjustment for differences in age, socioeconomic position in childhood, being born abroad, level of education, civil status, and history of unemployment before baseline reduces confounding from lifestyle-related risk factors in this study. As in other countries, it is known for Sweden that, for instance, low levels of education are associated with such risk factors (Falkstedt et al. 2016).

The main limitations of this study are due to its registry-based design. The use of secondary data sometimes leads to individual misclassifications, not only of the occupational exposures discussed above but also to misclassifications of individuals’ occupation, unemployment, and education level over time. The net effect of this on the presented associations is difficult to assess and, therefore, the associations should also be investigated using other study designs.

The regression adjustments could not include any direct measure of differences in health status before the follow-up period, but data on sickness absence history (minimum two weeks) among the individuals were available. However, we chose not to adjust for this, as disability pension among workers is almost always preceded by long-term sickness absence. A sensitivity analysis on those without any registered sickness absence the years before baseline did not indicate confounding from initial health status.

To conclude, in this study of middle-aged and older workers in Sweden, we found low levels of job control to be associated with higher risk of disability pension. Among the men, the association was dose–response shaped. Further, we found high levels of job demands to be associated with *lower* risk of disability pension across diagnosis categories among men but, among women, this lower risk did not hold for disability pensions with a psychiatric or a cardiovascular diagnosis. The association between low levels of job control and disability pension with a psychiatric diagnosis was found to be least affected in covariate-adjusted models and most similar between men and women. Whether modification of job demands, job control, and job strain can reduce middle-aged and older workers’ risk of disability pension needs to be investigated in future studies.

## Data Availability

Upon request.
